# HCK is a Potential Prognostic Biomarker that Correlates with Immune Cell Infiltration in Acute Myeloid Leukemia

**DOI:** 10.1155/2022/3199589

**Published:** 2022-03-04

**Authors:** Fang Cheng, Qiang Li, Jinglin Wang, Liu Wang, Weiming Li, Fang Zeng

**Affiliations:** ^1^Department of Pharmacy, Union Hospital, Tongji Medical College, Huazhong University of Science and Technology, Wuhan 430022, China; ^2^Hubei Province Clinical Research Center for Precision Medicine for Critical Illness, Wuhan 430022, China; ^3^Department of Hematology, Union Hospital, Tongji Medical College, Huazhong University of Science and Technology, Wuhan 430022, China

## Abstract

**Background:**

The tumor microenvironment (TME) plays a significant role in the progression and prognosis of acute myeloid leukemia (AML). This study is aimed at exploring TME-associated biomarkers and identify their potential mechanism in the microenvironment of AML.

**Method:**

In this study, the stromal, immune, and ESTIMATE scores of AML patients were evaluated with the ESTIMATE and CIBERSORT algorithms; then, the AML samples were divided into high- and low-score groups. We evaluated the association between clinicopathological characteristics, survival rate, and the stromal/immune/ESTIMATE scores. Furthermore, we identified TME-associated differentially expressed genes (DEGs) then carried out pathway enrichment analysis, protein-protein interaction (PPI) network, Cox regression analysis, and Kaplan-Meier survival analysis to select the most crucial genes. In addition, we further explored the potential mechanism of HCK in the AML microenvironment.

**Results:**

We identified 624 TME-associated DEGs and found that HCK was the most promising biomarker associated with AML. The results of the gene set enrichment analysis (GSEA) indicated that HCK was mainly involved in immune and inflammation-related signaling pathways. In addition, CIBERSORT analysis showed that HCK was closely related to tumor immune infiltration, with HCK expression associated with various infiltrating immune cells, including B cells, T cells, tumor-associated macrophages (TAM), NK cells, plasma cells, eosinophils, and neutrophils. Furthermore, HCK expression was closely related with ELN risk stratification in patients with AML.

**Conclusion:**

HCK could regulate immune cell infiltration in the microenvironment of AML and may act as a potential biomarker for the treatment and prognosis of AML patients.

## 1. Introduction

Acute myeloid leukemia (AML) is a highly heterogeneous hematological malignancy, which is the most common type of acute leukemia in adults [1]. Currently, the main treatment strategies for AML are intensive induction chemotherapy and postremission treatment. Although most patients with AML can achieve significant remission initially through chemotherapy, complete elimination remains a challenge [2]. Promising treatments have been proposed in recent years, such as CART cell therapy targeting CD33 and allogeneic hematopoietic stem cell transplantation [3, 4]; however, many patients are still at risk of disease recurrence and even die within 5 years after diagnosis. Therefore, identifying potential biomarkers will contribute to the diagnosis, treatment, and prognosis of patients with AML.

The tumor microenvironment (TME) has been considered a crucial factor for the diagnosis and response to treatment of cancer patients in recent years [5]. TME components (such as inhibitory immune cells, soluble factors, and extracellular matrix) could contribute to interfering with tumor immunotherapy, inducing chemoresistance, and promoting tumor progression [6]. Furthermore, the breakthrough approach of PD-1/PD-L1 targeted immunotherapy was found when evaluating tumor matrix interactions and TME-specific changes [7]. Therefore, the changes in the components of the TME may play a significant role in the entire process of malignant tumor progression and are considered to be important factors when identifying novel therapeutic targets. There are multiple and complex interactions among tumors, stromal cells, and immune cells due to changes in soluble factors and components of the extracellular matrix [8]. Stromal cells and infiltrating immune cells are two main nontumor components in the TME, which are related to the diagnosis and prognosis of tumor [9]. The interaction between leukemia cells and the bone marrow microenvironment has been shown to influence the chemoresistance of AML patients, which has become the focus of preclinical researches and clinical trials [10, 11].

In this study, we downloaded the gene expression profile data and clinical information from the TCGA-LAML database, and then, the immune and stromal scores of the AML patients were calculated using the ESTIMATE algorithm. Furthermore, we identified TME-associated differentially expressed genes (DEG) then carried out pathway enrichment analysis, protein-protein interaction (PPI) network analysis, Cox regression analysis, and Kaplan-Meier survival analysis to select the most crucial genes. Furthermore, we further explored the potential mechanism of the selected genes in the AML microenvironment. Our study may help clarify the important role of TME in AML and improve the prognosis and treatment of the patients of AML.

## 2. Methods

### 2.1. Data Source and Preprocessing

Firstly, we obtained mRNA expression and clinical data from the TCGA-LAML database. We removed patients with follow-up time <30 days and those lacking clinical information and finally obtained 200 patients. The mRNA expression data were processed with the R package and then calibrated, standardized, and log2 transformed. The ESTIMATE algorithm was used to calculate the “stromal score”, “immune score,” and “ESTIMATE score” in AML patients with the “estimate” package in R software. The stromal and immune score were calculated based on the relative proportion of the immune and stromal components. ESTIMATE scores were the sum of the two types of scores.

### 2.2. Subgroup Analysis of Clinicopathological Characteristics and Survival Analysis

AML patients were divided into high- and low-score groups according to stromal, immune, and ESTIMATE scores. We investigated the relationship between each score and the clinicopathological characteristics. In addition, Kaplan-Meier analysis was used to evaluate the association between the survival rate and the stromal/immune/ESTIMATE scores, which were tested by the log-rank test. The analyses were performed with the “survival” and “survminer” functions in the R software packages.

### 2.3. Identification and Enrichment Analysis of TME-Associated DEGs

DEGs in the stromal-score and immune-score groups were identified with the “limma” package in R software with the criteria of log2fold change (∣log2FC | >1) and false discovery rate (FDR) < 0.05. To study the biological function of these DEGs, Gene Ontology (GO) and Kyoto Encyclopedia of Genes and Genomes (KEGG) analyses were performed with “ggplot2,” “enrichplot,” and“clusterprofiler” package in R, and *P* < 0.05 was considered statistically significant. The GO analysis indicated the characteristics of DEGs in terms of biological process, cellular component, and molecular function [12], while the KEGG analysis reflected the enrichment of DEGs in the signaling pathways [13].

### 2.4. PPI Network and Cox Analysis for Screening the Most Important DEGs

We explored the correlation between these DEGs with a node association confidence score > 0.95 according to the STRING database (https://stringhttp://db.org/cgi/input.pl) [14]. Then, Cytoscape was applied to visualize networks. In addition, univariate Cox regression analysis was performed to identify candidate prognostic genes. The five most important genes (CD4, ITGAM, ITGB2, CCR5, and HCK) were finally obtained by cross-screening of the most connected PPI related genes and the candidate prognostic genes.

### 2.5. Evaluation the Prognostic Value of the Five Key Genes

Firstly, we compared the expression differences of these five genes between the normal group and the AML group. We then performed Kaplan-Meier survival analysis and log-rank test to evaluate the correlation between the five genes and overall survival (OS) using the R packages “survival” and “survminer”. In addition, Kaplan-Meier survival analysis was performed to investigate the relationship between prognostic genes and different clinical characteristics, including age and gender. The patients were divided into subgroups according to age (<65 years and ≥65 years), gender (male and female subgroups), and European LeukemiaNet (ELN) risk (favorable, intermediate, and poor prognosis).

### 2.6. Gene Set Enrichment Analysis

GSEA was used to evaluate the trend of gene distribution in a predefined gene set in a list of genes ranked by their relevance to the phenotype, thus determining their contribution to the phenotype [15]. GSEA analysis was conducted to investigate HCK-related signaling pathways in the high-expression group and the low-expression group. The enriched pathways were selected based on FDR < 0.05 after 1000 permutations.

### 2.7. Relationship between HCK and Immune Cell Infiltration

Based on the high specificity and sensitivity of gene expression profiles, CIBERSORT helps to distinguish 22 types of human immune cells, including T cells, B cells, macrophages, NK cells, dendritic cells, and myeloid subset cells [16]. To identify the association of HCK with tumor-infiltrating immune cells (TIICs), the CIBERSORT algorithm was used to evaluate the proportion of immune cells infiltrated in the TME. The relationship between TIICs and HCK was visualized by the “vioplot” package. Furthermore, a correlation analysis between HCK expression and TIICs was performed with the “limma,” “ggplot2,” “ggpubr,” “ggExtra,” and “corrplot” packages.

### 2.8. Statistical Analysis

Statistical analysis in this study was conducted by the R version 4.0.3. Group comparisons were performed with the *t*-test for continuous variables and *χ*^2^ − test for categorical variables. Spearman's or Pearson's correlation test was used for correlation analyses. The *P* value < 0.05 was considered a statistically significant difference.

## 3. Results

### 3.1. Correlation between Stromal, Immune, and ESTIMATE Scores with Overall Survival and Clinicopathological Characteristics in AML

**Figure 1:**
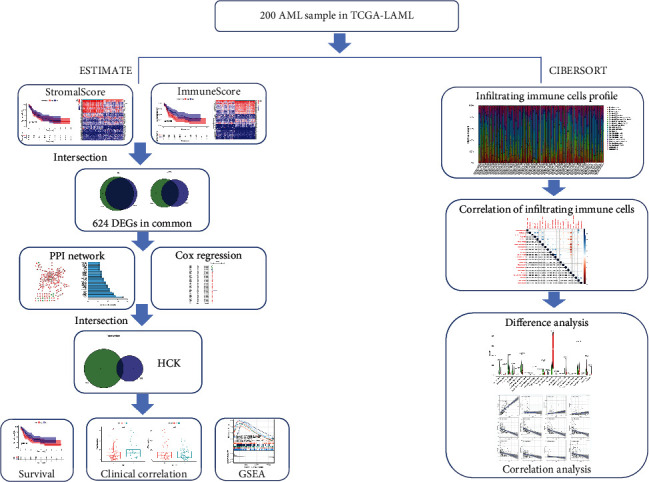
Work flow of the study.

**Figure 2:**
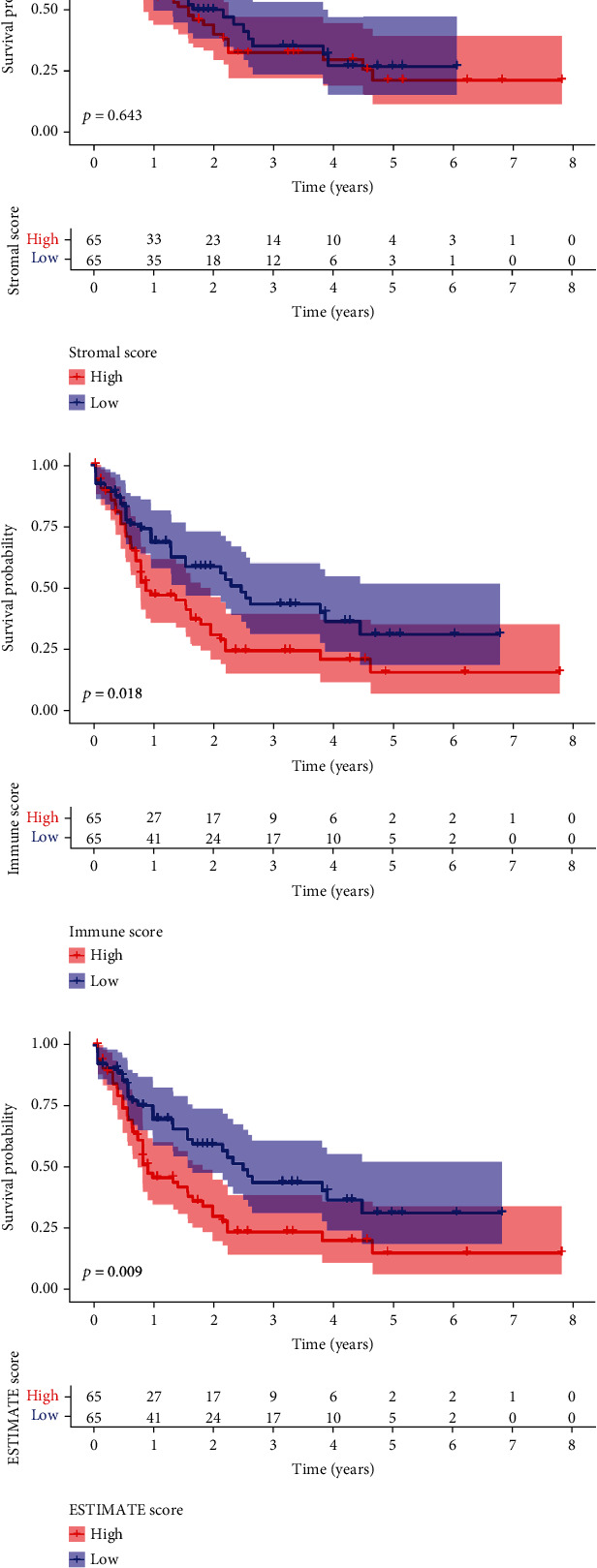
The correlation of the stromal, immune, and ESTIMATE scores with the overall survival rate of AML patients. (a) Stromal score group. (b) Immune score group. (c) ESTIMATE score group.

**Figure 3:**
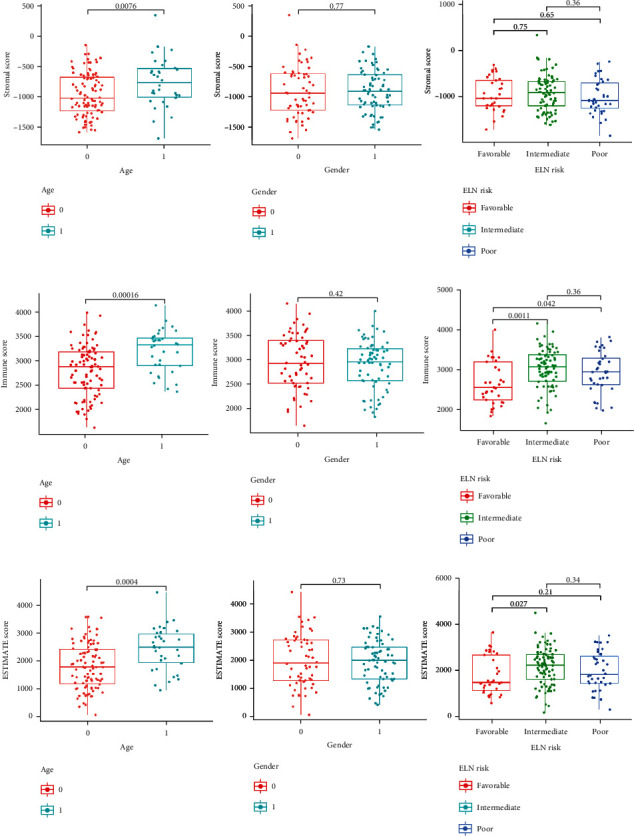
Correlation between scores and clinicopathological characteristics. (a) Correlation analysis of the stromal score, (b) immune score, and (c) ESTIMATE score with age, gender, and ELN risk stratification. 0 represents <65 years; 1 represents ≥65 years in the age subgroup; 0 represents male; 1 represents female in the gender subgroup.

The flowchart of the study was shown in Figure 1. The stromal, immune, and ESTIMATE scores for the AML patients were calculated using the ESTIMATE algorithm. Based on the scores, we assigned AML patients into the high- and low-score groups and compared the OS rates, respectively. The results showed that the low-score group had a better survival rate than the high-score group in the immune and ESTIMATE subgroups. However, no statistical differences were observed in the stromal subgroup (Figure 2). Furthermore, we also investigated the relationship between the stromal/immune/ESTIMATE scores and different clinical characteristics, including age, gender, and ELN risk. The patients were divided into subgroups according to age (<65 years and ≥65 years), gender (male and female subgroups), and European LeukemiaNet (ELN) risk (favorable, intermediate, and poor prognosis). The stromal/immune/ESTIMATE groups were closely associated with age; however, none of the scores was associated with the patients' gender. In the ELN risk subgroup, patients with high immune scores had a relativity poorer prognosis than those with low scores. Patients with an intermediate prognosis had higher ESTIMATE scores than those with a favorable prognosis but not significantly different from those with a poor prognosis. However, there were no statistical differences found in the stromal group (Figure 3).

### 3.2. Identification and Functional Enrichment Analysis of TME-Associated DEGs

**Figure 4:**
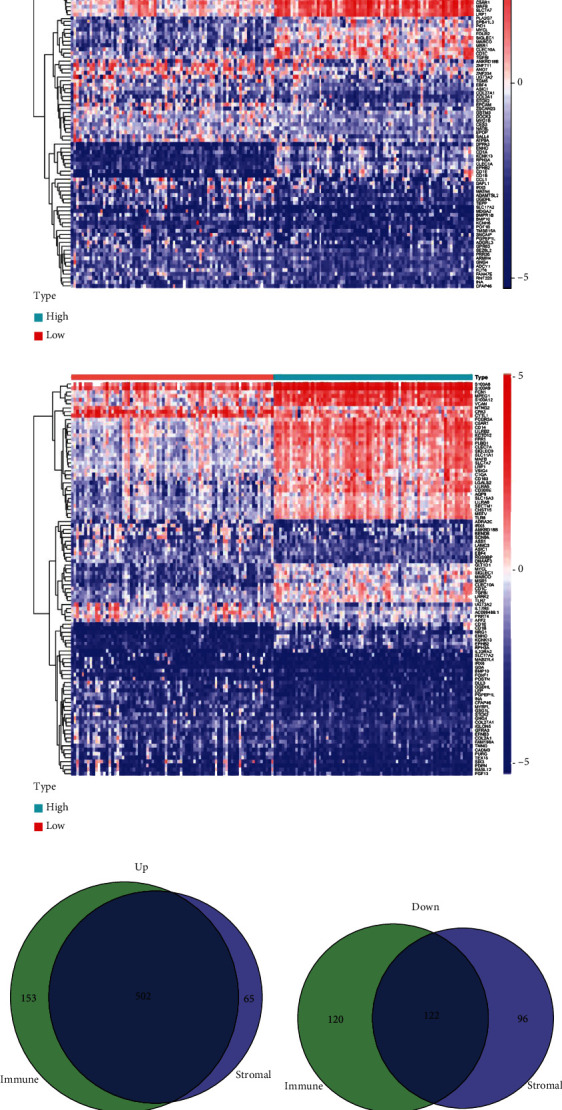
Identification of TME-associated differentially expressed genes (DEGs). (a) Heatmaps of top 50 DEGs between the high-score group and low-score group based on the stromal score and (b) immune score. (c) Venn diagram of common upregulated TME-associated DEGs and (d) common downregulated TME-associated DEGs.

**Figure 5:**
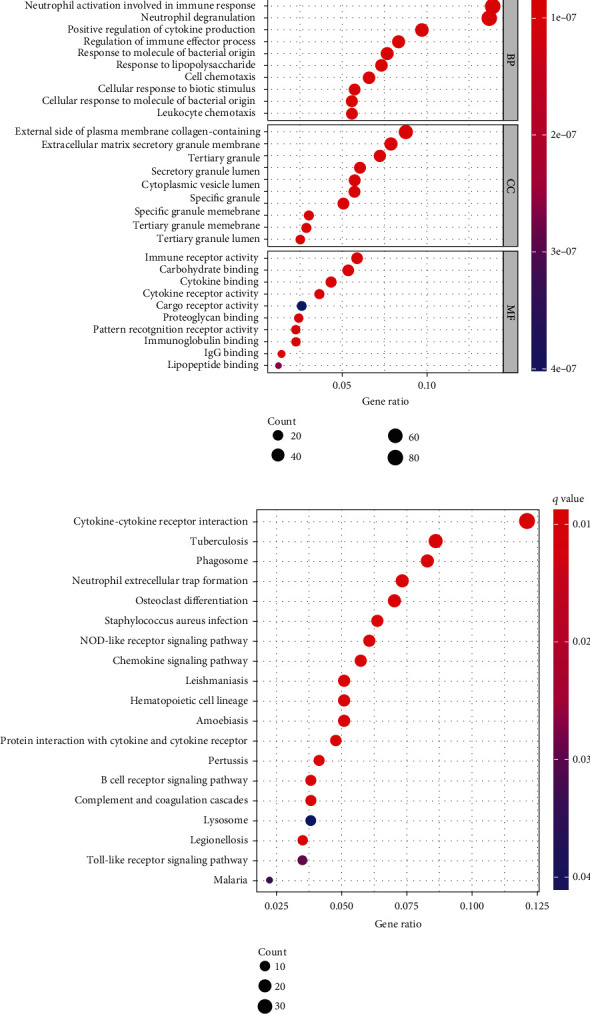
Functional enrichment analysis of common differentially expressed gene. (a) GO analysis. (b) KEGG pathway enrichment analysis.

Firstly, we conducted a differential analysis to identify the TME-associated DEGs and plotted the findings using a heatmap including the stromal and immune scores groups, respectively (Figures 4(a) and 4(b)). A total of 785 genes, including 567 upregulated genes and 218 downregulated genes, were identified as DEGs in the stromal group based on a threshold of *P* < 0.05 and ∣logFC | >1. Also, 785 DEGs were selected between the high-score and low-score stromal groups. In addition, 897 DEGs were identified in the immune group, including 655 upregulated and 242 downregulated genes. Finally, 624 common TME-associated DEGs were obtained, including 522 upregulated and 102 downregulated genes (Figures 4(c) and 4(d)). Then, the functional enrichment analysis was conducted to determine biological functions of the 624 TME-associated DEGs. The result of GO analysis showed that common DEGs were mainly involved in neutrophil activation involved in immune response, positive regulation of cytokine production, regulation of immune effector process, and immune receptor activity (Figure 5(a)). The KEGG analysis also showed that the TME-associated DEGs were significantly enriched in the cytokine-cytokine receptor interaction, the NOD-like receptor signaling pathway, and the chemokine signaling pathway (Figure 5(b)).

### 3.3. PPI Network and COX Analysis for Screening the Most Crucial DEGs

**Figure 6:**
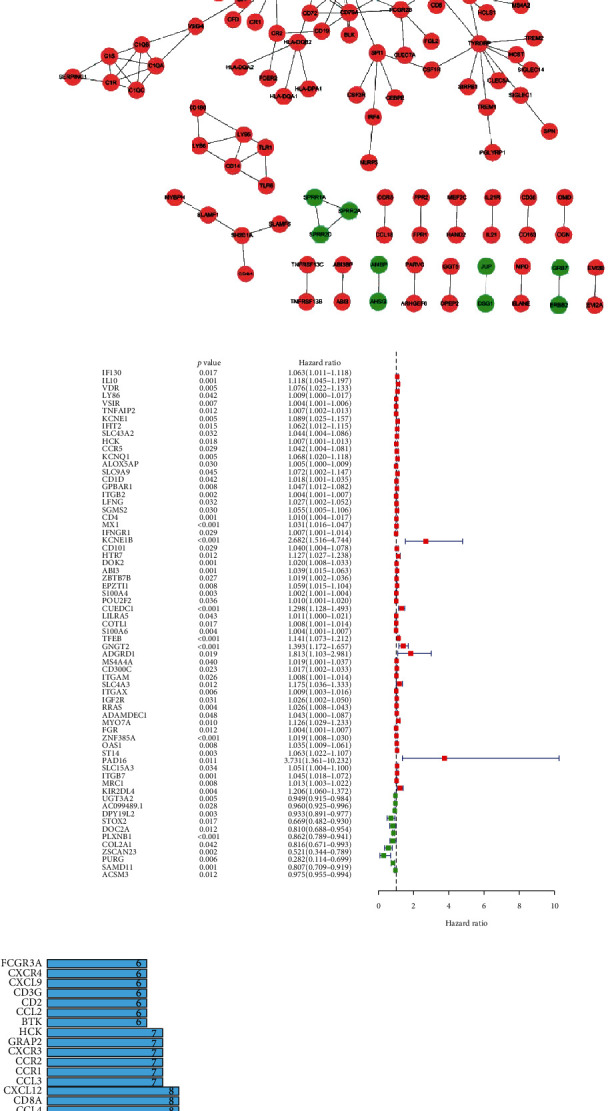
The intersection of the PPI network and univariate Cox regression analysis. (a) PPI network. (b) Top 30 differentially expressed genes screened from the PPI network. (c) Univariate Cox regression analysis. (d) Venn diagram of common DEGs shared by the top 30 genes from the PPI network and the prognostic genes in Cox regression analysis.

The PPI network consisted of 178 nodes and 288 edges (Figure 6(a)). The top 30 hub genes of the PPI networks were shown in Figure 6(b). A total of 67 prognostic DEGs were defined through univariate Cox regression analysis (Figure 6(c)). Then, an intersection analysis was performed between the top 30 hub genes and 67 prognostic DEGs, five crucial target genes (CD4, ITGAM, ITGB2, CCR5, and HCK) were finally screened out, and HCK was identified as a potential novel prognosis biomarker for AML patients. (Figure 6(d)).

### 3.4. Prognostic Significance of the Five Hub Genes

**Figure 7:**
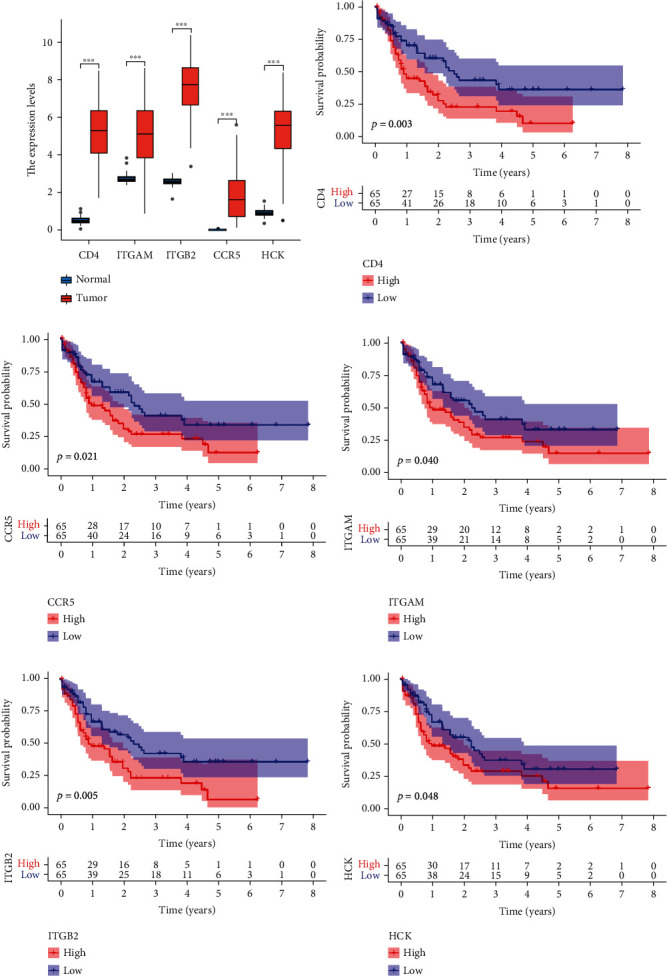
Prognostic significance of the five hub genes. (a) The expression differences of the five hub genes in normal and tumor samples. (b–f) Survival analysis.

**Figure 8:**
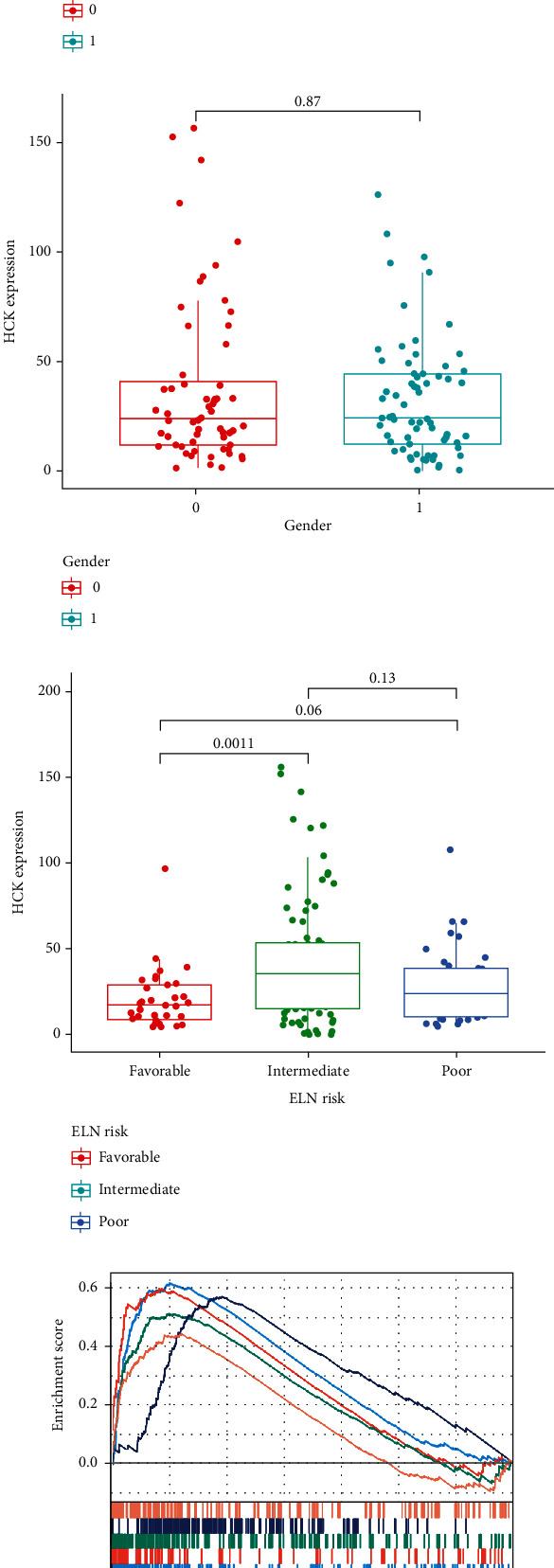
HCK was strongly correlated with prognosis in AML. The correlation of HCK expression with (a) age, (b) gender, and (c) ELN risk stratification. (d) GSEA analysis. 0 represents <65 years; 1 represents ≥65 years in the age subgroup; and 0 represents male; 1 represents female in the gender subgroup.

We further investigated the differences in expression of the five hub genes in normal and tumor samples. The results indicated that the five genes were highly expressed in the tumor sample compared to the normal tissues (Figure 7(a)). Then, we investigated the prognostic significance of the five hub genes in AML patients. These findings indicated that high expression of the five genes predicted a poor prognosis for patients with AML (Figures 7(b)–7(f)). In addition, we investigated the relationship between HCK expression and different clinical characteristics, including age, gender, and ELN risk stratification. The result indicated that HCK expression was closely associated with age, however, not related with gender. Patients with high HCK expression had a relatively poor prognosis, suggesting that HCK expression may be a promising predictor for AML patient prognosis (Figures 8(a)–8(c)).

### 3.5. GSEA Analysis

GSEA analysis was conducted to investigate HCK-related signaling pathways in the high-expression group and the low-expression group. The result indicated that HCK was mainly enriched in immune- and inflammation-related signaling pathways, including PI3K-AKT-MTOR signaling pathway, TNF*α* signaling via NF-*κ*B, poptosis, P53-pathway, and oxidative phosphorylation (Figure 8(c)).

### 3.6. CIBERSORT Algorithm

**Figure 9:**
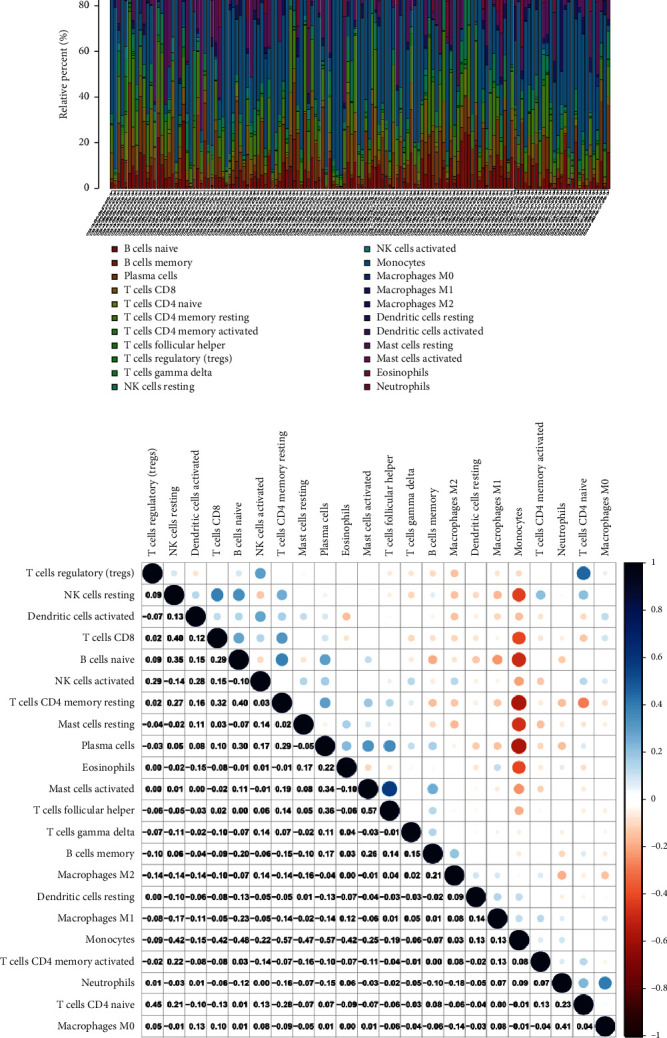
CIBERSORT algorithm. (a) The fractions of 22 types of tumor infiltratedimmune cells in AML. (b) Correlation with 22 types of tumor infiltrated immune cells.

The overall situation of TME immune infiltration in AML was shown in Figure 9(a). Each bar plot represented the proportion of 22 TIICs in each AML sample. Furthermore, the correlation between different TIICs in AML was shown in Figure 9(b). As shown in Figure 9(b), follicular helper T cells were positively correlated with activated mast cells (Cor = 0.57), while plasma cells, CD4 memory resting T cells, and monocytes were strongly negatively correlated (Cor = −0.57).

### 3.7. HCK Expression was Correlated with Immune Infiltration in AML

**Figure 10:**
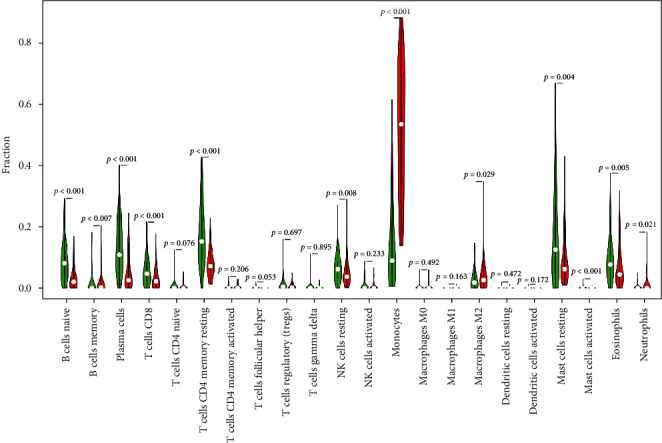
Comparisons of 22 types of tumor infiltrated immune cells between high and low HCK-expression groups.

We further explored the possible mechanism of HCK expression in TME, and correlation analysis between HCK expression and the TIICs was performed. The results showed that memory B cells, naive B cells, CD8 cells, resting CD4 memory cells, plasma cells, resting NK cells, monocytes, M2 macrophages, resting mast cells, activated mast cells, eosinophils, and neutrophils showed significant difference between the high and low HCK-expression groups (Figure 10).

### 3.8. Correlation Analysis of HCK Expression with TIICs in AML

**Figure 11:**
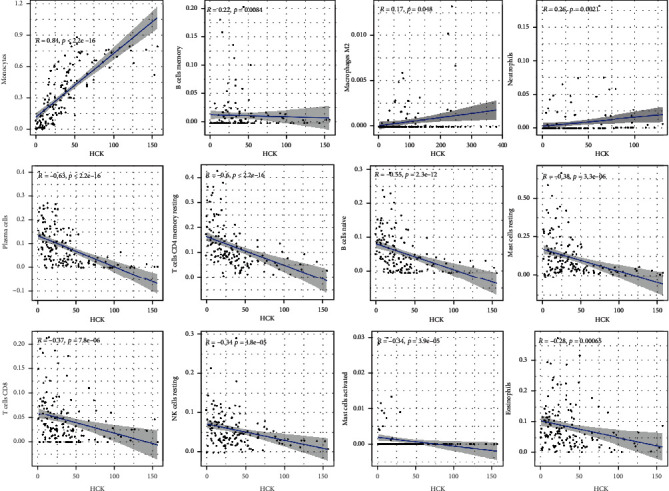
Correlation analysis between HCK and tumor-infiltrating immune cells.

We further investigated the correlations of HCK expression with TIICs in AML. The results indicated that HCK was positively correlated with the infiltration of monocytes (*R* = 0.84), memory B cells (*R* = 0.22), neutrophils (*R* = 0.26), and M1 macrophage (*R* = 0.17) but was negatively correlated with the infiltration of plasma cells (*R* = −0.63), resting memory CD4 cells (*R* = −0.6), native B cells (*R* = −0.55), resting mast cells (*R* = −0.38), CD8 cells (*R* = −0.37), resting NK cells (*R* = −0.34), activated mast cells (*R* = −0.34), and eosinophils (*R* = −0.28) (Figure 11).

## 4. Discussion

Recently, the TME has been proved to participate in the occurrence and progression of many cancers [17]. However, the specific mechanism and genes related to the progression and prognosis of AML in the TME remain to be determined. Therefore, exploring the interaction of stromal and immune cells in the TME may contribute to the development of novel therapeutic targets for AML. In our study, the stromal and immune scores of AML samples were evaluated with the ESTIMATE and CIBERSORT algorithms. The stromal, immune, and ESTIMATE scores were significantly associated with the clinicopathological parameters of AML, such as age and ELN risk. The results revealed that the stromal and immune cells that infiltrate the TME may play a crucial role in the progression of AML. We then performed enrichment analysis, PPI network, and Cox regression analysis to identify the most crucial TME-associated DEGs. Finally, five genes (CD4, ITGAM, ITGB2, CCR5, and HCK) were found to be associated with the OS of AML patients. Importantly, HCK was closely associated with immune infiltration and ELN risk stratification, which implied that HCK could be a promising novel therapeutic and prognosis target for AML through modulating the TME.

ITGAM is involved in the bone marrow differentiation and lysine specific demethylase-1 (LSD-1) activity, which contributes to the immune escape from leukemia cells [18]. The ITGAM also plays an important role in the tumor microenvironment in leukemia cell activation, chemotaxis, and cytotoxicity [19]. Several studies have shown that the positive expression of ITGAM was closely associated with the prognosis of AML patients [20, 21]. A previous study indicated that lncRNA ITGB2 was significantly associated with various immune signatures in AML [22]. Chemokines play an important role in tumor cell migration and infiltration of distant organ sites [23]. Natural human AML cells can generate CCL5 and express CCR5, and the CCL5/CCR5 axis can promote tumorigenesis by regulating the tumor microenvironment [24, 25]. HCK is a member of the Src family of nonreceptor tyrosine kinases, which is mainly expressed in myeloid and B lymphocyte cells [26]. Previous studies have indicated that HCK can regulate a variety of signal transduction pathways such as cell growth, proliferation, differentiation, migration, and apoptosis [27, 28]. Chemotherapy, targeted drugs, and hematopoietic stem cell transplantation are still the mainstays of AML treatment. Patients with AML show great heterogeneity in clinical manifestations and treatment prognosis; thus, adequate risk stratification prior to treatment is particularly important for choosing a reasonable treatment path [29]. The ELN risk classification was widely used for risk stratification of patients with AML [30]. Therefore, we further investigated the relationship between HCK expression and ELN risk stratification. The result indicated that patients with high HCK expression had a relativity poor prognosis, suggesting that HCK was closely associated with the development and prognosis of patients with AML.

With the rapid development of immunotherapy for AML in recent years, including agents targeting CTLA-4 and PD-1, research on key components in the TME has gradually become a hot topic. Therefore, we investigated the potential influence of HCK on the TME. The result of GSEA findings indicated that HCK was enriched in immune and inflammatory-related pathways, suggesting that HCK could be involved in regulating immune activity in the AML microenvironment. Furthermore, we evaluated the proportion of infiltrated immune cells in the TME with CIBERSORT algorithm. Then, the correlation analysis between HCK and infiltrated immune cells in the TME was further estimated with difference and correlation analysis. The results indicated that HCK was associated with a variety of infiltrated immune cells, including B cells, T cells, NK cells, monocytes, plasma cells, mast cells, tumor-associated macrophages (TAMs), and neutrophils.

Previous studies have determined that inhibition of HCK can target TAMs, which leads to the reduction of infiltrated immune cells, lessened immunosuppression, and an improved efficacy for chemotherapy pancreatic ductal adenocarcinoma [31]. In addition, inflammatory mediators secreted by the tumor cell immune microenvironment can induce HCK activation in macrophages and neutrophils, promoting tumor expansion and invasion [32–34]. Furthermore, HCK also plays a crucial role in neutrophil phagocytosis [35]. Therefore, in addition to its direct carcinogenic effects on leukemia cancer cells, early tumor cells can stimulate excessive activation of HCK in adjacent immune cells by promoting cytokine secretion, to strengthen the role of tumor-promoting microenvironment [28, 36]. Take together, these results imply that HCK may regulate immune cell infiltration in the AML microenvironment and is expected to be a potential biomarker for the treatment and prognosis of AML patients.

However, this study has some limitations. Firstly, the TME score of tumor tissue was calculated based on the ESTIMATE algorithm and the proportion of immune cells was performed with the CIBERSORT algorithm based on mRNA expression data. However, more real-world optimization is required, as these algorithms are still in the exploratory stage. Furthermore, an external cohort is lacking to validate the role of the TME-score in predicting treatment response in the clinical setting. In addition, all the results were obtained from bioinformatics analysis and cannot be validated due to the absence of experimental findings. Therefore, future experimental verification is required to clarify the potential mechanisms of HCK in the AML microenvironment.

## 5. Conclusion

In this study, five genes (CD4, ITGAM, ITGB2, CCR5, and HCK) were found to be related to the OS of AML patients. Importantly, HCK was closely associated with immune infiltration and ELN risk stratification, which implied that HCK might be a novel promising therapeutic and prognosis target for AML duo to its modulatory activity in the TME.

## Data Availability

The datasets analyzed during the current study are available from the corresponding author Fang Zeng (fancyzeng@126.com) on reasonable request. The data that support the findings of this study are available at the TCGA data portal (https://tcga-data.nci.nih.gov/tcga/).

## Abbreviations

TME: Tumor microenvironment
AML: Acute myeloid leukemia
DEGs: Differentially expressed genes
ELN: European LeukemiaNet
PPI: Protein-protein interaction
GSEA: Gene set enrichment analysis
FDR: False discovery rate
TAM: Tumor-associated macrophages
OS: Overall survival
TIICs: Tumor-infiltrating immune cells
TCGA: The cancer genome atlas
GO: Gene Ontology
KEGG: Kyoto Encyclopedia of Genes and Genomes
CD4: Cluster of differentiation 4
ITGAM: Integrin alpha-M
ITGB2: Integrin beta-2
CCR5: C-C chemokine receptor type 5
HCK: Hemopoietic cell kinase.
